# Deep Learning Framework for Real-Time Estimation of *in-silico* Thrombotic Risk Indices in the Left Atrial Appendage

**DOI:** 10.3389/fphys.2021.694945

**Published:** 2021-06-28

**Authors:** Xabier Morales Ferez, Jordi Mill, Kristine Aavild Juhl, Cesar Acebes, Xavier Iriart, Benoit Legghe, Hubert Cochet, Ole De Backer, Rasmus R. Paulsen, Oscar Camara

**Affiliations:** ^1^Physense, BCN Medtech, Department of Information and Communication Technologies, Universitat Pompeu Fabra, Barcelona, Spain; ^2^DTU Compute, Technical University of Denmark, Kongens Lyngby, Denmark; ^3^IHU Liryc, University Hospital of Bordeaux, Bordeaux, France; ^4^Department of Cardiology, Rigshospitalet, University of Copenhagen, Copenhagen, Denmark

**Keywords:** geometric deep learning, left atrial appendage, convolutional neural network, thrombus-atrial fibrillation, computational fluid dynamics, principal component analysis

## Abstract

Patient-specific computational fluid dynamics (CFD) simulations can provide invaluable insight into the interaction of left atrial appendage (LAA) morphology, hemodynamics, and the formation of thrombi in atrial fibrillation (AF) patients. Nonetheless, CFD solvers are notoriously time-consuming and computationally demanding, which has sparked an ever-growing body of literature aiming to develop surrogate models of fluid simulations based on neural networks. The present study aims at developing a deep learning (DL) framework capable of predicting the endothelial cell activation potential (ECAP), an *in-silico* index linked to the risk of thrombosis, typically derived from CFD simulations, solely from the patient-specific LAA morphology. To this end, a set of popular DL approaches were evaluated, including fully connected networks (FCN), convolutional neural networks (CNN), and geometric deep learning. While the latter directly operated over non-Euclidean domains, the FCN and CNN approaches required previous registration or 2D mapping of the input LAA mesh. First, the superior performance of the graph-based DL model was demonstrated in a dataset consisting of 256 synthetic and real LAA, where CFD simulations with simplified boundary conditions were run. Subsequently, the adaptability of the geometric DL model was further proven in a more realistic dataset of 114 cases, which included the complete patient-specific LA and CFD simulations with more complex boundary conditions. The resulting DL framework successfully predicted the overall distribution of the ECAP in both datasets, based solely on anatomical features, while reducing computational times by orders of magnitude compared to conventional CFD solvers.

## 1. Introduction

Atrial fibrillation (AF) is the most common clinically significant arrhythmia, with a cumulative lifetime development risk above 30% in individuals of European ancestry (Benjamin et al., 2019). AF is defined by a quivering or irregular heartbeat (arrhythmia) caused by chaotic electric activity, which leads to irregular contraction and wall rigidity of the left atrium (LA), preventing effective flow of the blood to the ventricles. Such hemodynamic alterations, alongside factors such as endothelial or endocardial dysfunction, including a state of hypercoagulability, increase the risk of cerebrovascular accidents by allowing thrombus formation within the LA (Watson et al., 2009); if dislodged, such thrombi can occlude the cerebral circulation, causing a thromboembolic (ischemic) stroke. In fact, non-valvular AF is responsible for 15–20% of all cardioembolic ischemic strokes, which preferentially form at the left atrial appendage (LAA) (Cresti et al., 2019), an heterogeneous, tubular structure derived from the anterior wall of the LA.

In this regard, researchers have explored the correlation between LAA morphology and the risk of stroke (Yaghi et al., 2020; Dudzińska-Szczerba et al., 2021; Słodowska et al., 2021). Nonetheless, so far the results have been ambiguous, as the current classifications and associated morphological parameters of the LAA are often entirely subjective, hand-crafted features; there is a need for more systematic shape analysis of the LAA with advanced and observer-independent computational tools such as statistical atlases (Slipsager et al., 2019).

Besides, due to the critical role of blood stasis in thrombogenesis, the interest in the analysis of LA hemodynamics is gaining momentum. Yet, the intricate behavior of the left atrium as a modulator of left ventricular filling (reservoir, conduit, and booster pump function; Vieira et al., 2014), coupled to a substantial anatomical heterogeneity, makes modeling left atrial hemodynamics a notoriously difficult task. Consequently, computational fluid dynamics (CFD) analyses have emerged as an invaluable tool in analyzing the mechanistic relationship between patient-specific organ morphology and blood stasis (García-Isla et al., 2018; Masci et al., 2019; García-Villalba et al., 2021). Nevertheless, conventional CFD methods are renowned for their large memory requirements and long computing times (Liang et al., 2018), which also involve extensive pre-processing of each patient-specific mesh, resulting in studies with very limited sample sizes and severely hindering its suitability for time-sensitive clinical applications.

As a response, neural networks have increasingly been employed in complex dynamical systems such as fluid dynamics, resulting in highly accurate surrogate models that can be evaluated with significantly less computational resources and several orders of magnitude faster than conventional finite element solvers (Hennigh, 2017). Recently, deep learning (DL) has made its way into biological fluid modeling, aiming at predicting blood velocity vector fields or derived hemodynamic parameters that play a crucial role in the diagnosis and development of several cardiovascular diseases (Liang et al., 2018; Li et al., 2021). Nevertheless, most studies have mostly focused on structures such as the aorta, which present a less complex morphology and hemodynamic profile than the LA and LAA. That being said, applying conventional DL models to non-Euclidean domains, such as graphs and meshes, in which medical data is often best represented, is not a trivial task, as most widespread neural networks can only operate over regular data such as images (Fey et al., 2018). In this regard, geometric deep learning approaches, which are tailored to operate over graph data, have already been applied to biomedical meshes, especially in cardiac electrophysiological models (Grandits et al., 2021; Meister et al., 2021).

Hence, in the present study, we have leveraged a collection of distinct DL models, which are well-tailored to deal with mesh data, to develop a CFD surrogate capable of learning the complex relationship between the heterogeneous LAA morphology and the endothelial cell activation potential (ECAP), parameter linked to an increased risk of thrombosis. By employing neural networks, there is no need to manually craft morphological features, ensuring that the model only learns the most relevant anatomical characteristics toward the automatic prediction of ECAP. Moreover, once trained, neural networks allow the prediction of ECAP maps in new unseen patients, orders of magnitude faster than it is possible with current CFD solvers. The implemented DL approaches included principal component analysis (PCA) based shape analysis coupled to fully connected layers, flattening the LAA morphology to a UV space to leverage convolutional neural networks (CNN) and geometric deep learning, which is perfectly suited to non-Euclidean data such as meshes. All the mentioned methods were first tested on a simplified LA model containing 256 real and synthetic LAA (dataset 1). In addition, the best performing model was further tested on a second, more realistic 114 patient dataset, which incorporated the entire patient-specific LA anatomy (dataset 2).

## 2. Methods

**Figure 1:**
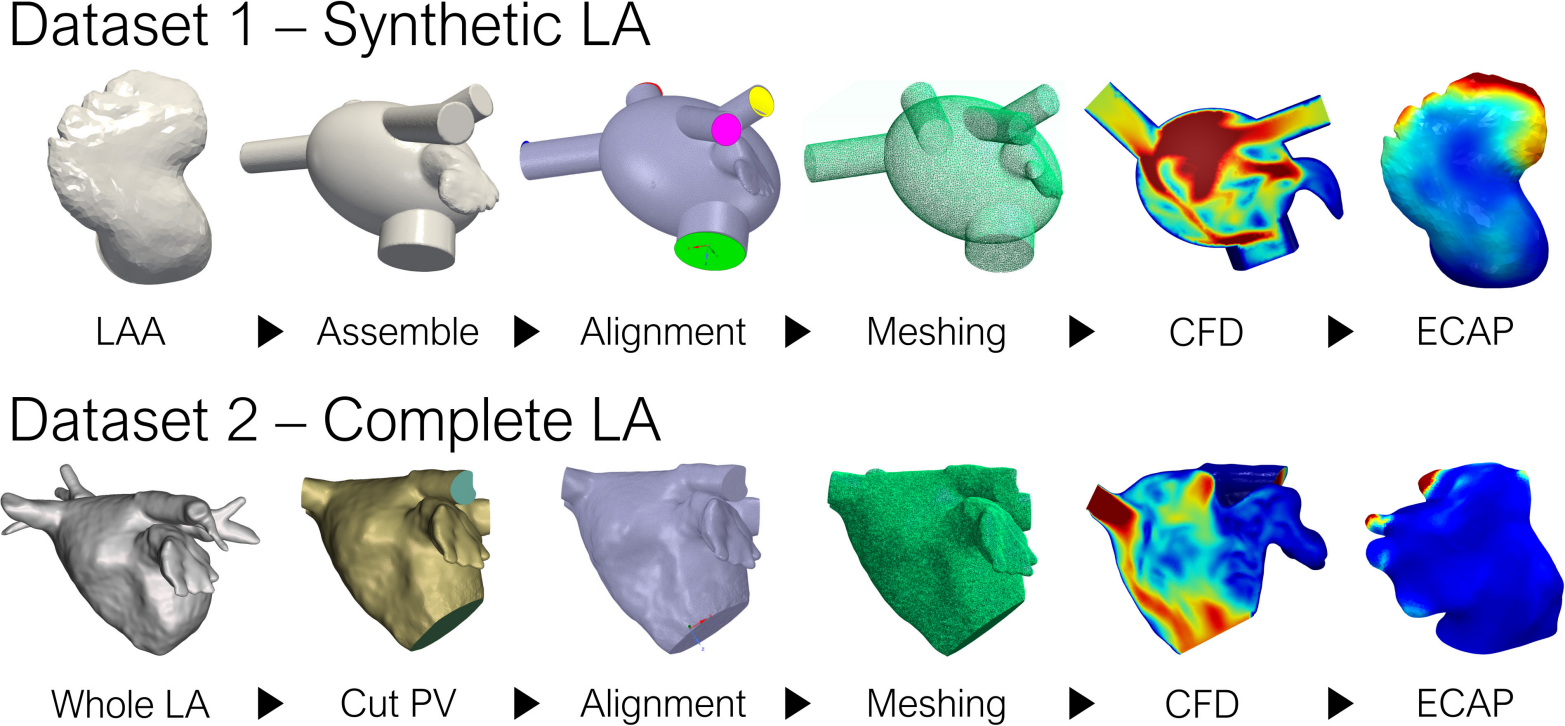
Pipeline to generate the ground truth ECAP maps for the two datasets. LA, left atrium; LAA, left atrial appendage; PV, pulmonary veins; CFD, computational fluid dynamics; ECAP, endothelial cell activation potential.

The overall pipeline employed to generate the ground-truth data (i.e., the *in-silico* ECAP index from CFD simulations in the whole dataset of 370 geometries) is shown in Figure 1. Preprocessing slightly differed between the two datasets: LAA comprising dataset 1 were all assembled to an oval LA, while in dataset 2, which considered the whole patient-specific LA anatomy, all pulmonary veins (PV) were trimmed at the first branching to define the inlets and outlets. Later, tetrahedral volumetric meshes were generated to run the CFD simulations and compute the ground truth ECAP maps. For the networks to capture the most relevant morphological features, the triangular meshes employed to describe the anatomy of each LAA had to be transformed according to the prerequisites of each of the implemented DL methods. Lastly, the neural networks were trained to learn the arbitrary non-linear function linking the geometry of the LAA and its corresponding ECAP maps. Model prototyping and fine-tuning were completed on the synthetic LA dataset (dataset 1). Afterward, the best-performing network was further tested in the more realistic, complete LA dataset (dataset 2).

### 2.1. Data

The first dataset (dataset 1) was derived from computed tomography (CT) images provided by the Department of Radiology at Rigshospitalet (Copenhagen, Denmark) acquired as part of the Copenhagen General Population Study (Nordestgaard et al., 2012). It was comprised of 256 LAA, combining 54 real patients and 202 synthetic LA geometries. The latter, being borrowed from a preceding study (Morales et al., 2020), stem from a statistical shape model (SSM) based on 103 real LAA surfaces (Slipsager et al., 2019). In this synthetic LA model, only the geometry of the LAA was considered, as incorporating the highly heterogeneous LA anatomy would qualitatively increase the inter-subject variability of the hemodynamic parameters. Thus, all appendages were assembled to a common oval approximation of the LA (García-Isla et al., 2018), reducing the complexity of the maps to be predicted, and ensuring that the LAA morphology remained the only independent variable affecting the ECAP values.

Conversely, the second dataset considered the complete patient-specific LA morphology during CFD simulations (dataset 2). The data was provided by Hospital Haut-Lévêque (Bordeaux, France), originating from pre-procedural high-quality CT scans from 114 AF patients that underwent a left atrial appendage occlusion (LAAO) intervention. Both studies were approved by the local Institutional Ethics Committees, and all patients provided informed consent.

### 2.2. CFD Simulations

A total of 370 CFD simulations were run to generate the ground-truth data, 256 of which corresponded to the synthetic LA dataset, while the remaining 114 were part of dataset 2. All synthetic LAA simulations on dataset 1 were borrowed from a preceding study, adjusting the setup of the simulations for the remaining 54 real morphologies accordingly (Morales et al., 2020). First, an input velocity profile was set in the PV, based on clinical observations (Fernández-Pérez et al., 2012). Second, the mitral valve (MV) was considered as a wall boundary during diastole, while an outlet pressure of 1,067 Pa was set through systole. The motion of the LA was based on a diffusion-based dynamic mesh emanating from the MV ring plane, adjusted according to literature (Veronesi et al., 2008; Mill et al., 2019). Only a single heartbeat was completed for each simulation.

On the other hand, simulations from dataset 2 featured more complex boundary conditions (BC), with the inlet being defined at the PV based on pressure wave measurements from an AF patient; the velocity outlet was set on the MV based on Doppler ultrasound velocity profiles derived from a single patient. Therefore, while the LA morphology was completely patient-specific, the same boundary conditions were shared among all cases. However, all BCs were synchronized to their corresponding patient's electrocardiogram. The dynamic mesh governing LA motion was changed to a spring-based model. Unlike in dataset 1, three full heartbeats were completed for each simulation, aiming to reach a steady state. Only the last heartbeat was considered when computing the risk indices of thrombosis. Lastly, whereas final tetrahedral volumetric meshes for dataset 1 consisted of ~350 k elements, each mesh from dataset 2 doubled that figure at around 800 and 900 k elements, after a mesh convergence study that included meshes up to 1 M elements.

Simulations were computed on Ansys Fluent 19 R32 (ANSYS Inc,USA)^1^ and automatized leveraging the MATLAB AAS toolbox,^2^ while post-processing was performed in Paraview^3^ alongside in-house python scripts. The blood was treated as a Newtonian fluid, with a density of 1,060 Kg/m^3^ and a viscosity of 0.0035 Pa/s, while using time-steps of 0.1 s.

The endothelial cell activation potential (ECAP), proposed by Di Achille et al. (2014), was the parameter chosen to evaluate the risk of thrombosis in the LAA. Since the pathophysiology of thromboembolism in AF is based upon the formation of mural thrombi, the ECAP focuses on hemodynamic behavior in the proximity of the vessel wall. More precisely, the ECAP is defined as the ratio between the oscillatory shear index (OSI) and the time-averaged wall shear stress (TAWSS).

(1) $$\begin{array}{r} ECAP=\frac{OSI}{TAWSS}. \end{array}$$

Thereby, a dimensionless parameter related to thrombosis is obtained, thus, avoiding the need for a more complicated neural network architecture capable of handling temporal sequential data. High ECAP values result from low TAWSS and high OSI values, indicating the presence of low velocities and high flow complexity, which is associated with endothelial susceptibility and risk of thrombus formation.

### 2.3. Deep Learning Architectures

#### 2.3.1. Principal Component Analysis—Fully Connected Model

**Figure 2:**
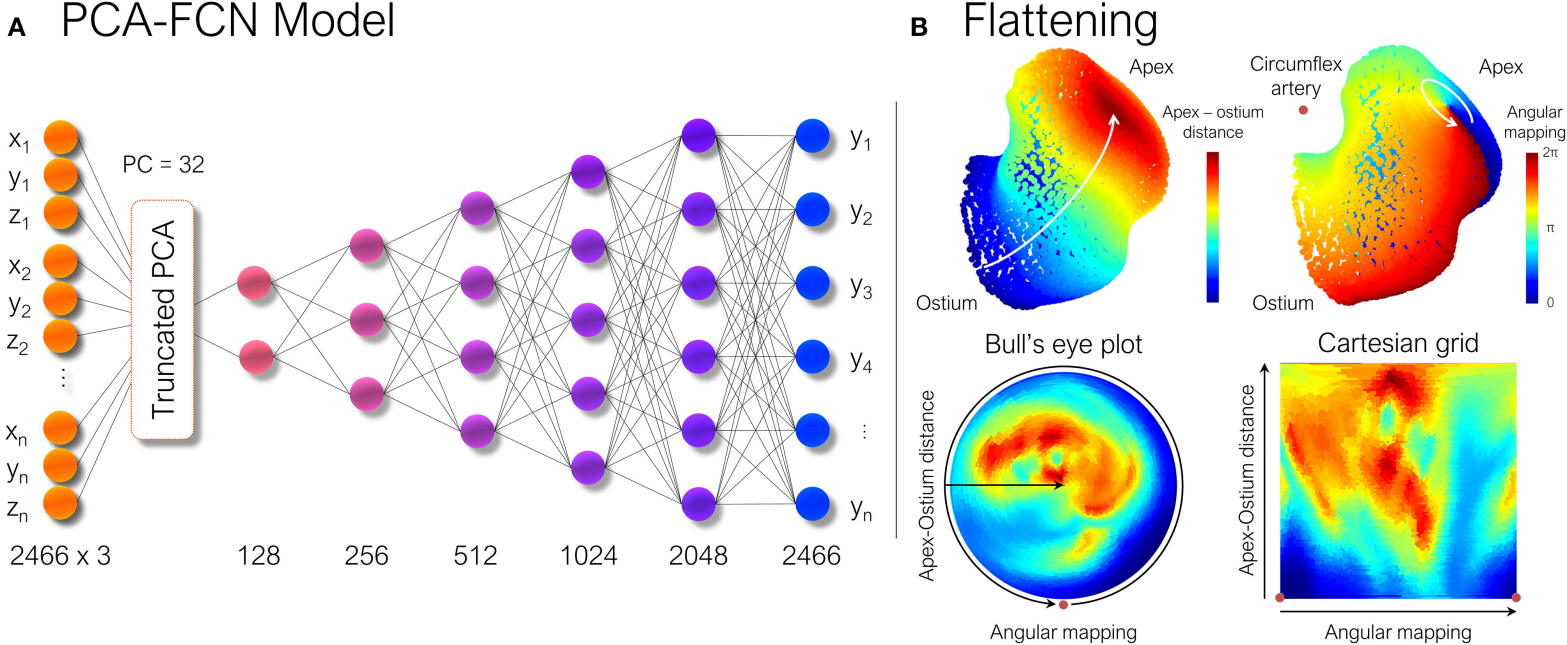
**(A)** Scheme of the principal component analysis model (PCA). Once each shape was parameterized, the ECAP values were predicted through a simple multilayer perceptron formed by fully connected layers (FCN). Numbers below each layer represent the number of nodes in each of them. **(B)** The LAA morphology is flattened to a new 2D UV space in which the new axes are represented by the geodesic apical-ostium distance and the angle formed with respect to the centroid of each isoline, using the closest point to the circumflex artery as reference. ECAP, endothelial cell activation potential.

Although it is gradually being replaced by more sophisticated non-linear models, principal component analysis (PCA), has long been employed to learn a linear latent space of 3D registered meshes, for tasks such as compression, reconstruction, and animation (Zhou et al., 2020). Nevertheless, PCA requires all meshes to be registered to a common template so that the same topology and connectivity are shared among them. In our case, this step was completed through non-rigid volumetric registration of signed distance fields, based on the work by Slipsager et al. (2019), registering all meshes to a common template comprised of 2,466 vertices. For the PCA analysis, the spatial coordinate of the nodes composing the mesh were employed as the input features. Thus, the morphology of each LAA can be expressed by a small set of scalar values through truncated PCA following:

$$\begin{array}{r} X=\bar{X}+\overset{M}{\underset{i=1}{\sum }}\alpha_{i}\sqrt{\lambda_{i}}W_{i},\;\to \;\alpha_{i}=\frac{W_{i}^{T}\left(Y-\bar{Y}\right)}{\sqrt{\lambda_{i}}},\text{(2)} \end{array}$$

where $\bar{X}$ is the mean shape, *W*_*i*_ and λ_*i*_ being the set of eigenvectors and eigenvalues of the covariance matrix for the retained number, *i*, of principal components (PC). Hereby, if the variability of the dataset is explained by a small set of PCs, each LAA anatomy can be expressed by a number *i*, of α_*i*_ scalars that can be fed directly to any regular neural network. For our dataset, a total of *n* = 32 PCs were kept, provided that the training dataset was large enough. By doing so, 97.6% of the morphological variability was retained for the synthetic cases and 94.1% for the real LAA geometries. Afterward, the non-linear mapping between the low dimensional representation of LAA morphology and its corresponding ECAP maps was completed through a fully connected feed-forward neural network (FCN). It comprised five hidden layers, as shown in Figure 2A, sequentially increasing the size of each layer. The whole model was implemented in Keras,^4^ using TensorFlow^5^ as backend.

#### 2.3.2. UV Mapping—U-Net Model

Although PCA models have been extremely successful (Tewari et al., 2017), they often struggle to capture finer details, since the resulting latent space is a linear combination of the input features. Alternative models such as convolutional neural networks (CNN) are widely employed to capture spatial features in regular grids (Zhou et al., 2020), which owing to a combination of desirable properties, such as local connectivity, weight sharing, and displacement invariance, became the backbone of fields such as computer vision. That being said, due to the irregular nature of mesh data, spatially-shared convolution kernels cannot be directly leveraged, unless the 3D mesh data is mapped to a UV space, also known as flattening.

**Figure 3:**
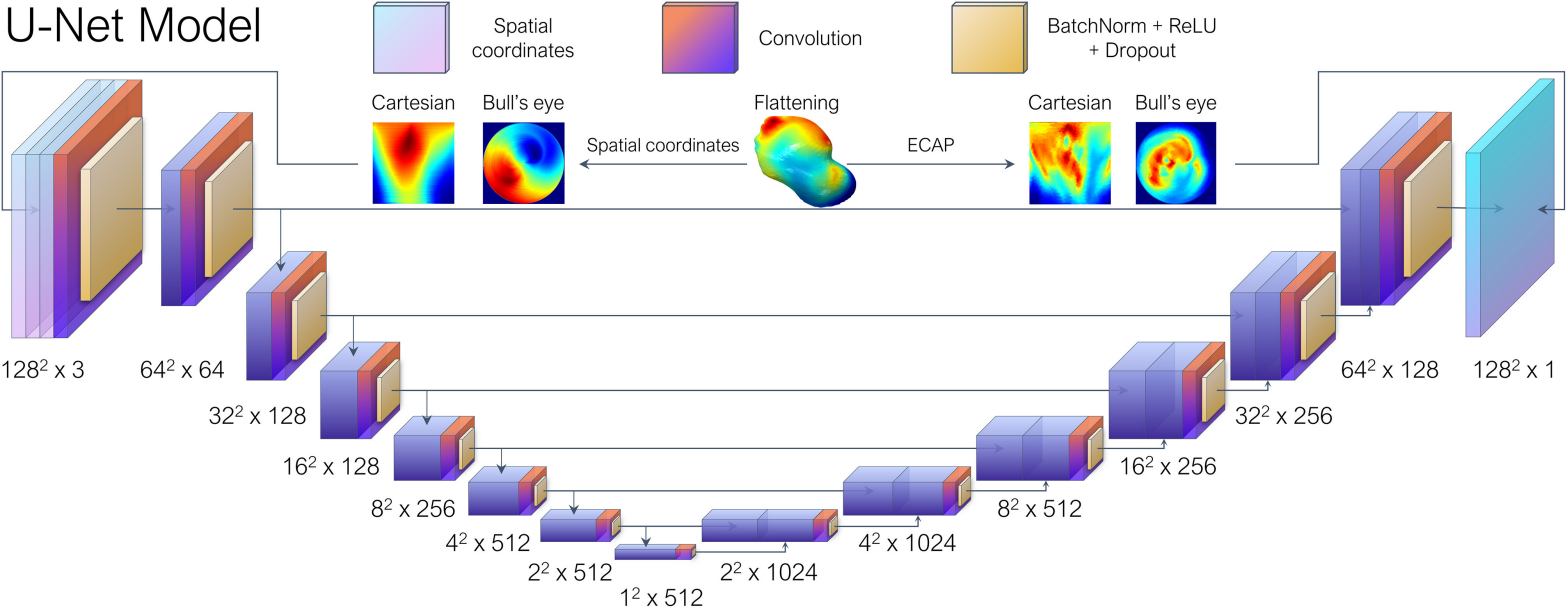
Each left atrial appendage (LAA) went through UV mapping, more colloquially known as flattening, and represented as a 2D image either as Bull's eye plot or a polar coordinate-based Cartesian grid representation. The U-Net architecture based on the work by Thuerey et al. (2020), received the spatial coordinates of the vertices sampled during the flattening process as the input features and then performed regression to predict their corresponding ECAP maps.

Consequently, the LAA were “flattened” based on the approach described in Acebes et al. (2021). First, each LAA was divided into an equivalent number of isolines, based on the geodesic distance from the ostium to the LAA apex, which was computed through a heat equation method (Crane et al., 2013). Subsequently, an equivalent number of vertices were sampled from each isoline, through an angular mapping performed by pivoting around the centroid of each isoline. Meanwhile, the points closest to the position of the circumflex artery, which was manually marked from the CT images, were chosen as the reference 0–360° angle. Once polar coordinates had been derived, each LAA mesh was represented as a 2D image either as a circumferential polar plot, also known as a Bull's eye plot (Cerqueira et al., 2002), with the apex of the LAA located in the center of the circumference (see Figure 2B), or as a rectangular image whose two axes consist in the apical-ostium distance and angular mapping. Even though the outer ring of the bull's eye plot undergoes distortion, it better preserves the LAA topology avoiding the cut-off produced by the flattening process. Conversely, the Cartesian grid representation faces much stronger warping in the area close to the apex, which is far overrepresented relative to its actual surface area in the 3D mesh. Therefore, both flattened representations were included to weigh up their trade-offs. Lastly, as shown in Figure 3, the bull's eye plot was padded to a rectangular image before feeding it to the neural network. During training, the padded regions were not taken into account when performing the loss calculation and subsequent accuracy measurements.

With regards to the DL model, we opted for a conventional U-Net architecture which is comprised of overlapping convolutional layers arranged in a typical encoder-decoder bowtie structure (Ronneberger et al., 2015), consisting in sequential pooling operations that ensure that multi-scale features are learned from the input data. In addition, skip connections, encourage the network to reuse low-level features in the decoding layers, which result in state-of-the-art performance in several tasks such as medical segmentation. The vanilla U-Net provided by Thuerey et al. (2020) was leveraged, which was implemented to predict turbulent flow over a set of distinct airfoils. In our case, the input features consisted in the spatial coordinates of the vertices sampled from each LAA mesh during UV mapping, arranged as a three-channel depth tensor, analogous to an RGB image in computer vision tasks. Similarly to the original paper, seven convolutional blocks were employed (Figure 3), each including batch normalization layers and ReLU activations after convolution layers, as is the standard practice.

#### 2.3.3. Geometric Deep Learning

While UV mapping enables the direct leveraging of CNNs over meshes, the resulting 2D image suffers from distortion as the original mesh data must be cut and warped. Moreover, it cannot be easily extended to more general volumetric data. Whilst alternative workarounds such as voxelization exist, the most efficient way of representing 3D surface shapes and topologies is through polygonal meshes (Hanocka et al., 2019). As a response, a set of emerging methods, under the umbrella term of Geometric DL, are succeeding in generalizing DL models to non-Euclidean domains such as polygonal meshes, seamlessly extending operations such as convolutions to the native form of the data (Bronstein et al., 2017).

Among the array of available graph CNN layers, we opted for SplineCNN (Fey et al., 2018), since being a spatial method, it offers several advantages when dealing with meshes. In particular, it avoids the need of establishing mesh correspondence. Additionally, defining the spatial relations between vertex features becomes trivial by employing pseudo-coordinates. In our use case, pseudo-coordinates were obtained by computing the relative distance in Cartesian coordinates between the vertices of each edge. During the training process, these edge attributes define how the input features will be aggregated in the neighborhood of a given node. Additionally, the vertex-wise curvature and normals were fed to the network as vertex features.

**Figure 4:**
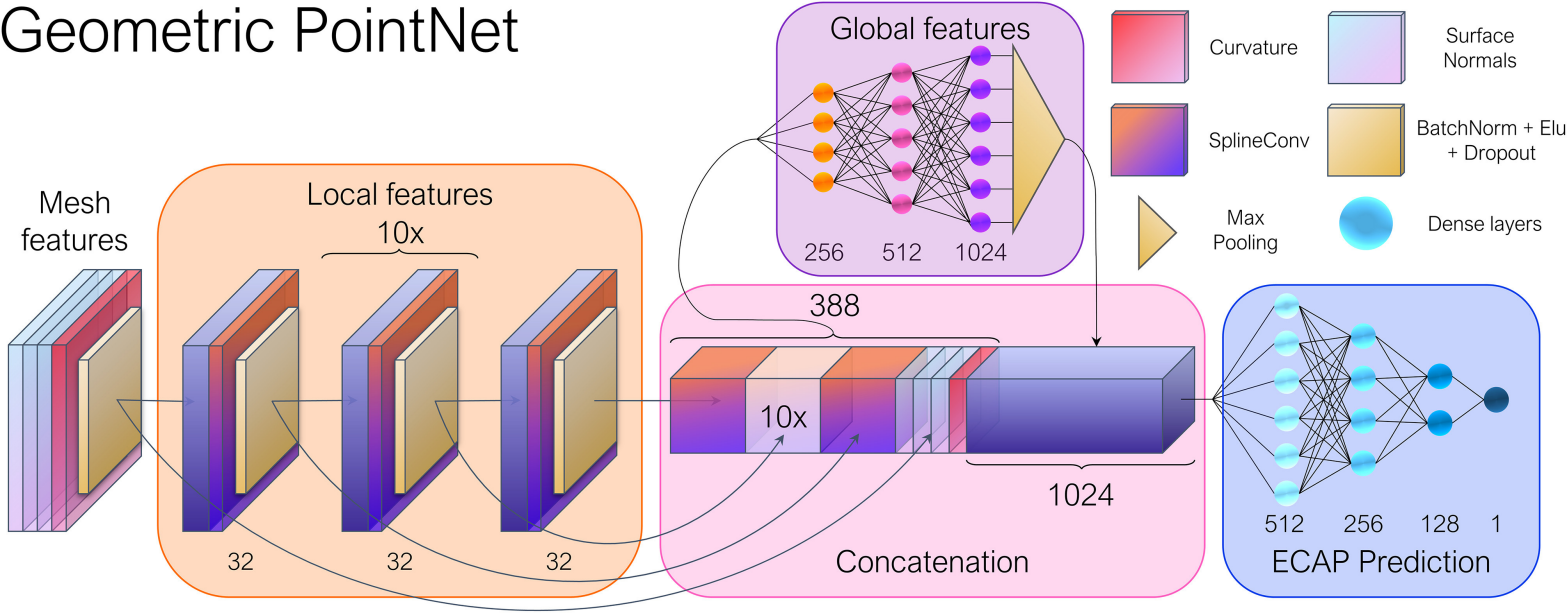
General overview of the geometric deep learning network architecture. The input vertex features consisted of the point-wise curvature and normal vectors. The spatial relations between the nodes were stored as edge attributes through Cartesian pseudo-coordinates. Twelve consecutive SplineConv layers (Fey et al., 2018) were employed in the local feature module, while a 1,024 feature vector was obtained after max pooling, representing the global features of each mesh. Numbers adjacent to each layer indicate the number of output features.

As aforementioned, besides convolution, operations such as pooling and strided convolutions play a key role in the success of CNNs, by allowing the network to sequentially extract larger scale and abstract features. Consequently, a PointNet-inspired (Qi et al., 2016) architecture was implemented, in which a series of consecutive layers focus on learning local features. Subsequently, the resulting feature arrays are concatenated and fed to a multilayer perceptron that generates a vector of global features using max-pooling, as shown in Figure 4. An almost identical model was employed by Meister et al. (2021) to estimate left ventricular depolarization times, albeit using a distinct convolutional operator. By swapping the multilayer perceptrons employed on the original PointNet (Qi et al., 2016) for graph convolutional layers, we expect to better exploit local correlations and weight sharing, while providing topological information to the network, which is explicitly absent in point cloud data.

The model was constructed by using PyTorch Geometric (PyG),^6^ a Geometric DL extension of PyTorch.^7^ PyG offers a broad set of convolution and pooling operations that extend the capabilities of traditional CNNs to irregularly structured data such as graphs and manifolds. With this in mind, the mesh dataset resulting from the simulations were converted into individual graphs. Together with PyVista,^8^ we converted each mesh to a graph represented by *G* = (*V*, *E*), with V=1,…,N being the set of nodes, and E corresponds to the set of edges of the triangular faces. For each vertex, we computed the curvature and surface normal vectors, totaling four input feature channels.

### 2.4. Hyperparameter Tuning

A thorough grid search was carried out to fine-tune the models by iteratively swapping several hyperparameters while keeping a fixed seed in the dataset split. In the PCA-FCN and U-Net model, ReLU activations were employed coupled with a learning rate of *lr* = 0.01 and *lr* = 0.0005 and trained during 150 and 300 epochs, respectively. Concerning general hyperparameters of the geometric DL model, the exponential linear unit (ELU) provided the best results among all activation functions, as it is standard in many mesh-related tasks. In addition, the training loop was carried out through 300 epochs with a batch size of 16 and a learning rate of 0.001. In regards to the parameters of the SplineConv layer, a B-spline basis of degree 1 and a kernel size of *k* = 5 were chosen, following suggestions by the authors (Fey et al., 2018). All models employed a dropout of 0.1 and included Adam as the optimizer. Similarly to Thuerey et al. (2020), while alternative loss functions such as L_2_ Loss and smooth-L_1_ yielded similar results, the L_1_ loss still had an edge over them.

### 2.5. Experimental Setup

All of the above-presented models were first tested on the synthetic LA dataset (dataset 1). Some of the experiments aimed to determine whether the synthetic data resulting from the statistical shape model were sufficiently representative of real patient data. If so, synthetic data could be of help with the data-hungry nature of neural networks in the face of data scarcity, which is a recurring issue in the medical field. On the one hand, k-fold cross-validation was performed, first training in the real (*n* = 54) and synthetic (*n* = 202) cases separately, and later combining both datasets. We have called these experiments “Cross real,” “Cross synth,” and “Cross,” respectively. The experiment in the real dataset was divided into 6-folds while the latter two experiments run 8-folds to ensure the groups were even. In addition, as only the areas of high ECAP values are pro-thrombotic, we wanted to assess the capability of the models in predicting the areas with the highest ECAP. For this purpose, a binary classification was performed taking the 90*th* percentile of the distribution as the threshold, which roughly equated to 4, following a similar approach to Di Achille et al. (2014) in abdominal aortic aneurysms.

On the other hand, the second set of experiments was conducted in which the amount of training data was sequentially scaled, to monitor the generalization and accuracy improvement (or lack thereof) on the testing dataset. Therefore, the testing scheme from the cross-validation experiments was maintained, but several runs were completed for each testing fold, changing the amount of available training data on each. For the first two experiments, which we deemed “Sequential Real” and “Sequential synthetic,” the real and synthetic morphologies were trained and tested separately. These experiments aimed to learn which models performed better with few amounts of data. Alternatively, in a third experiment, all the 202 synthetic LAA were employed as the training baseline. On top of this baseline, real geometries were sequentially added while testing on the remaining real cases. The objective of this experiment, deemed as “Sequential real+synthetic,” focused on the number of real cases required to build a model just trained on synthetic data, being able to properly generalize to patient-specific LAA morphologies. In all the aforementioned experiments, 10% of all the training data was employed as validation and used to select the best performing model. In addition, due to the stochastic nature of the training process in neural networks, the presented results have been averaged across several runs.

Lastly, the best-performing model from the previous experiments underwent further testing on dataset 2. While the complete patient-specific LA morphology was included during simulation, solely the LAA anatomy was fed to the neural network during the prediction of the ECAP maps. A single 10-fold cross-validation experiment was completed in this dataset along with the binary classification. The 90th percentile equalled 16 in this case.

## 3. Results

The ECAP distributions resulting from both simulations were distinct due to the different geometry and boundary conditions. The ECAP maps from the LA synthetic dataset had a mean value of μ = 2.14 ± 1.41, whereas dataset 2 exhibited a far more lopsided distribution, with a mean value of μ = 34.82 ± 251.68 but a median equal to 0.492.

Each simulation in the synthetic LA dataset lasted around 3–4 h, whereas it took at least 24 h to complete every single dataset 2 simulation, some requiring up to 48 h. Conversely, the PCA model was the fastest training DL network by a long margin, only requiring an average of 2 min to train. The training runs of the remaining two networks (i.e., UV mapping—U-net and Geometric DL) by contrast, lasted around 15–20 min. Once the models were trained, the prediction of ECAP maps pertaining to new unseen cases was instantaneous. On the other hand, the graph-based network was the lightest, with a total of 1.686.097 trainable parameters, in comparison to the 7.846.178 weights in the PCA model, and 9.304.833 for the U-Net.

The accuracy results for the cross-validation experiments are provided in Table 1, both in terms of the mean absolute error (MAE) of the ECAP and the true positive error (TPR), that is, the percentage of areas above the 90th percentile that have been predicted as such by the network. The geometric DL network outperformed the remaining approaches in all cross-validation experiments for both metrics. Nonetheless, there is a noticeable disparity between the MAE and classification results, given that even though the cross-validation on real data has provided accuracy on par to the other two scenarios in terms of MAE, it has a significantly lower TPR among all models.

**Table 1:** Prediction accuracy results in terms of mean absolute error (MAE) and true positive rate (TPR) for the cross-validation experiments.

**Model**	**Cross**	**Cross real**	**Cross synth**	**Cross (%)**	**Cross real (%)**	**Cross synth (%)**
	**Mean absolute error (MAE)**			**True positive rate (TPR)**		
PCA-FCN	0.608 ± 0.021	0.591 ± 0.023	0.603 ± 0.008	69	41	69
Cartesian	0.651 ± 0.007	0.661 ± 0.028	0.617 ± 0.017	55	20	64
Bull's eye	0.654 ± 0.009	0.582 ± 0.027	0.628 ± 0.009	64	33	65
Geometric	**0.521** **±0.013**	**0.519** **±0.021**	**0.514** **±0.017**	**77**	**57**	**79**

*PCA, principal component analysis; FCN, fully connected network. Bold signals the model with the best value for a given experiment and metric*.

**Figure 5:**
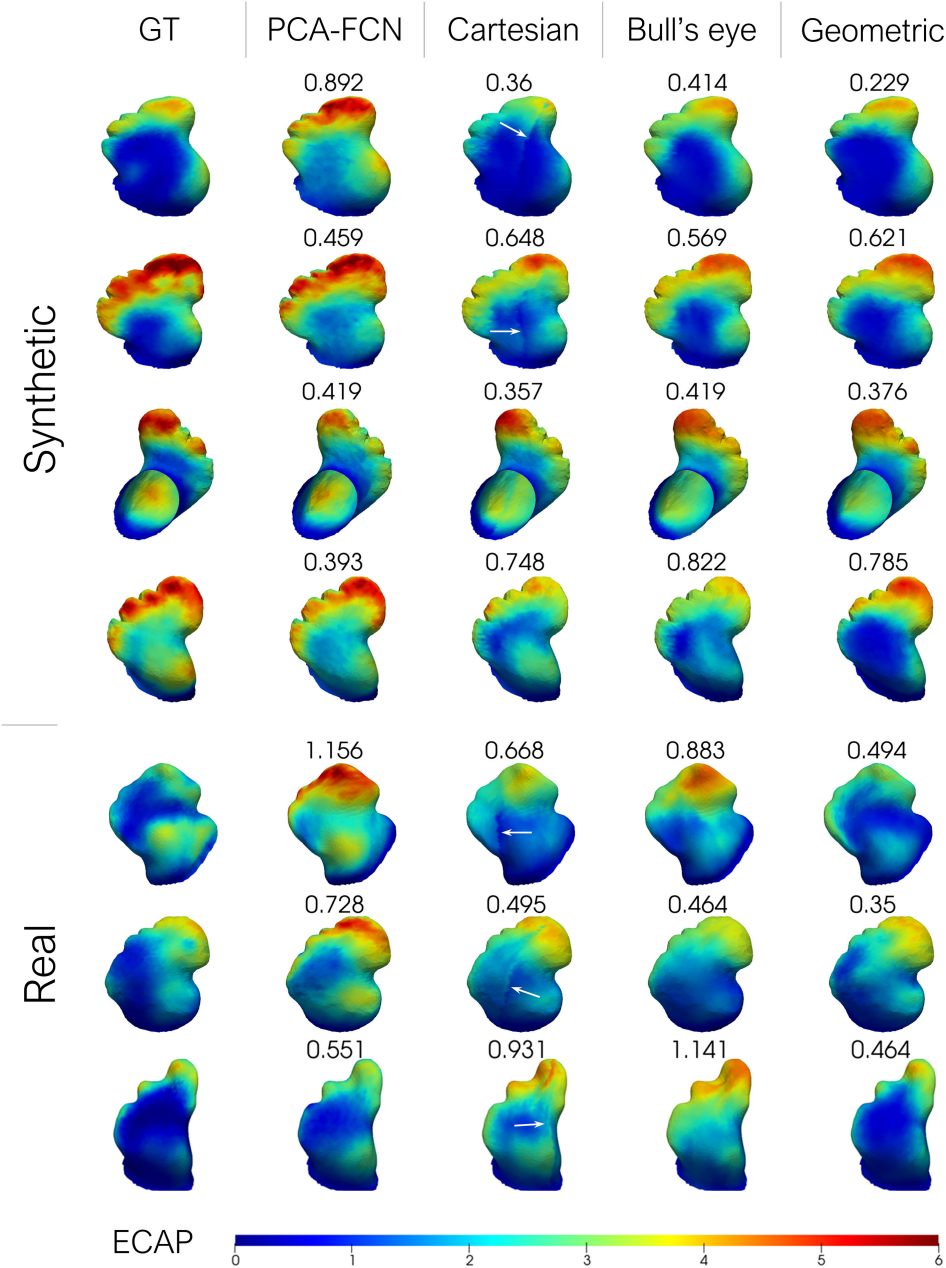
From left to right: ground-truth endothelial cell activation potential (ECAP) from fluid simulations; principal component analysis model (PCA-FCN) prediction; Cartesian grid and Bull's eye plot prediction; geometric deep learning prediction (Geometric). The mean absolute error (MAE) is also provided alongside. Higher ECAP values (in red) are linked to a higher risk of thrombus formation.

Additionally, a small batch of seven testing geometries from one random fold of the “Cross” cross-validation experiment is shown in Figure 5. Cases in rows 1–4 of the figure were derived from the SSM model while the remaining three represent real patient cases. The results from the remaining test samples in the fold are provided as Supplementary Material. In order to visually compare with the rest of the approaches, the results derived from the flattening models were interpolated back to the original mesh. Furthermore, the mean absolute error is provided for each prediction instance.

**Figure 6:**
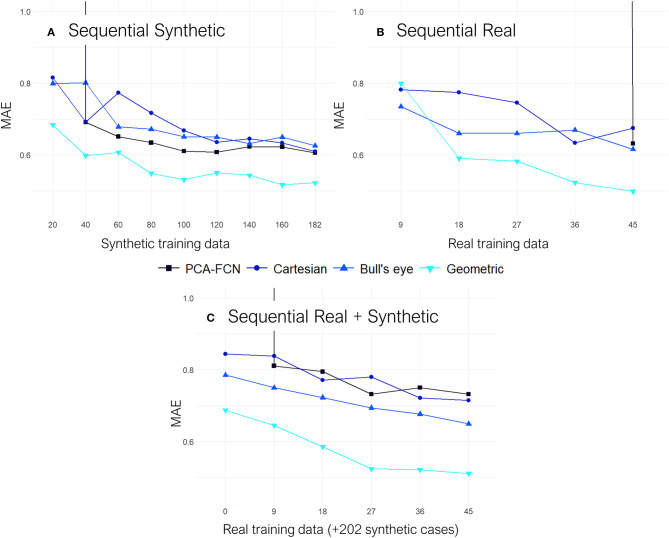
All results are shown on terms of the mean absolute error (MAE). **(A)** Results from the “Sequential Synthetic” experiment in which only the synthetic data was employed for training. **(B)** Results from the “Sequential Real” experiment which only trained and tested on the real cases. **(C)** Results from the “Sequential Real + Synthetic” test, in which all the synthetic geometries were employed as the training baseline and subsequently, batches of real data were sequentially added on top.

In Figure 6, the results from the sequential experiments are provided. Once again the graph-based model outscored its counterparts by some margin. Conversely, the PCA model struggled whenever few data was available as the maximum number of PCs had to be lowered in folds with <32 training instances. Interestingly, the bull's eye representation also had an edge over the rectangular Cartesian grid in the majority of tasks. Furthermore, the addition of synthetic cases in the training dataset for experiment “Sequential Real + Synthetic,” did not improve upon the results of models solely trained on real data for the “Sequential Real” experiment.

**Figure 7:**
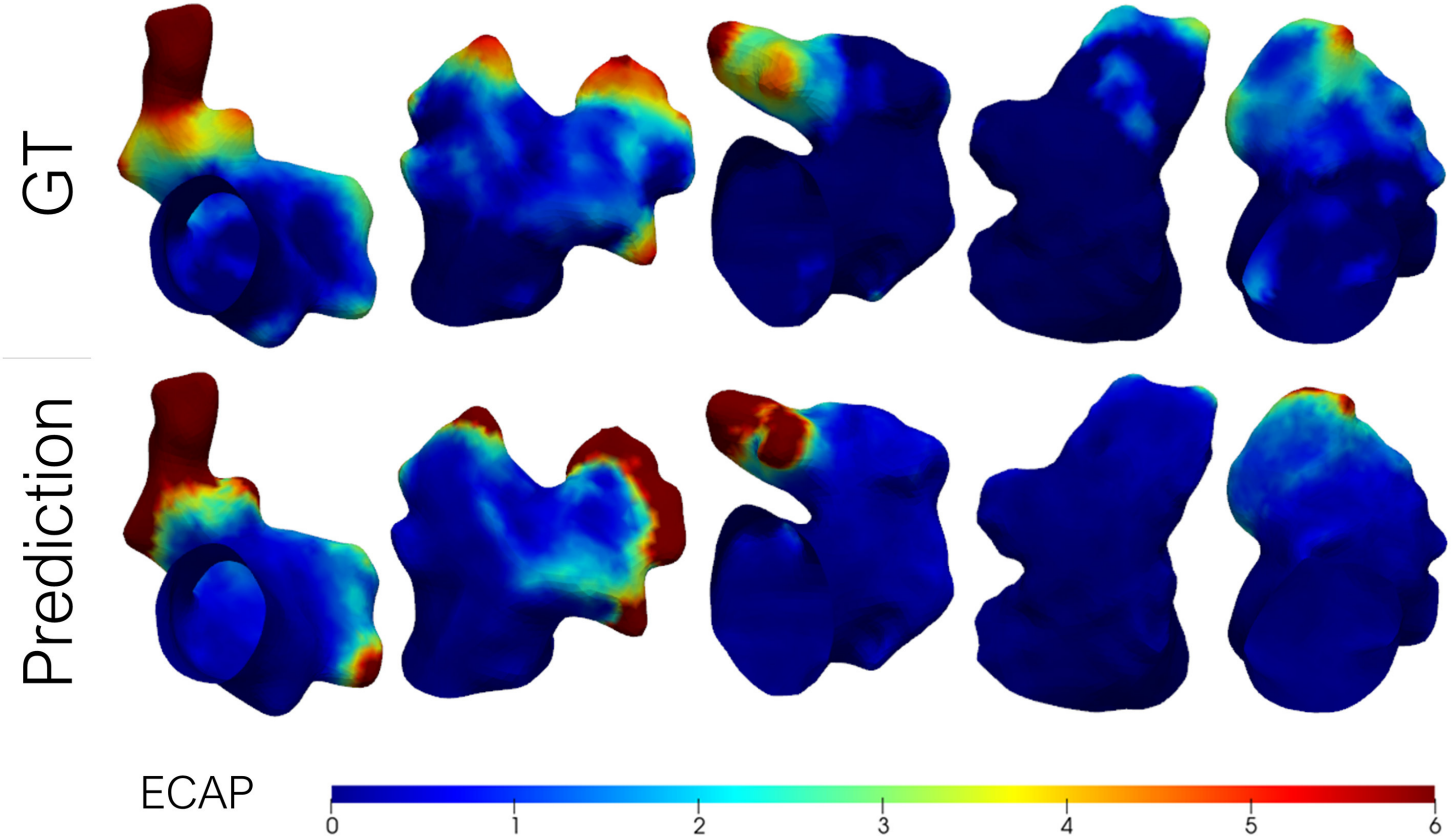
Predicted endothelial cell activation potential (ECAP) maps in a batch of left atrial appendages (LAA) from dataset 2 (i.e., patient-specific LA) alongside the ground-truth (GT) from fluid simulations. Higher ECAP values (in red) are linked to a higher risk of thrombus formation.

Lastly, some exemplary results from the Geometric PointNet on the more complex dataset 2 are showcased in Figure 7. The remaining test subjects are provided as Supplementary Material. The cross-validation resulted in a MAE = 1, 506±0, 543, while a TPR of 70% was achieved on the binary classification task.

## 4. Discussion

The primary goal of this work was to accurately estimate the ECAP, an *in-silico* thrombosis risk index, with a set of distinct deep learning approaches, thus, being able to instantaneously predict the risk indices when presented with new morphologies, without the need of running time-intensive and computationally demanding simulations. It is evident from Figures 5, 7 that the developed framework, especially the one based on geometric DL, successfully mimicked the behavior of two distinct sets of CFD simulations with different boundary conditions, managing to capture the global ECAP distributions solely on the basis of LAA morphology. Moreover, the geometric DL model seamlessly extends to realistic data without the need for template registration or 2D mapping. More importantly, the training and prediction of the *in-silico* index were completed orders of magnitude faster than conventional solvers of fluid simulations (i.e., tens of minutes vs. several hours). Furthermore, once trained inference can be completed instantaneously.

In this regard, a proper understanding of the data to be learned by the network was imperative in simplifying the space of results and achieving good accuracy. In our case, proper scaling of the data turned out to be crucial in improving the results. For instance, the input tensor to the U-Net model, containing the spatial coordinates of the vertices was standardized. Similarly, power transforming of the curvature data in the graph neural network also offered superior performance. The clearest example, however, involved the ECAP maps obtained from dataset 2 (e.g., with patient-specific LA data) which had a very marked positive skew compared to dataset 1 (e.g., with synthetic LA), rendering the model completely unable to learn. This issue was easily resolved by *log* transforming the ECAP maps for training, resulting in an almost symmetrical data distribution which could then be reconstructed back to visualize the results. The resulting distribution densities are provided as Supplementary Material.

By far, the most laborious and time-consuming aspect of the study consisted in setting up and running the 370 CFD simulations. Several of the steps typically involved in a geometry-specific fluid modeling pipeline (i.e., medical image segmentation, mesh building, the definition of boundary conditions, simulation execution), often necessitate manual intervention (e.g., mesh corrections). This lack of automatization represents a major bottleneck when simulating large datasets, hence most fluid dynamics studies end up including <10 cases when focused on complex morphologies such as the LA and LAA. By automating several of the aforementioned procedures we managed to streamline most of the simulation workflow, thus enabling the formation of a dataset large enough to train neural networks.

### 4.1. Dataset 1—Synthetic LA Dataset

Careful inspection of the results presented in Table 1 indicates that the geometric DL model outperformed all its counterparts in the three designated tasks. A look at Figure 5 further supports this hypothesis, as the geometric DL network obtains a better accuracy than the rest of the models in 5 out of 7 of the shown cases. Interestingly, despite having a more rudimentary DL architecture, the PCA model was the best non-geometric DL approach, even when trained on the real cases alone. An strong performance of the PCA network on the synthetic dataset was to be expected since the geometries were sampled from a statistical shape model based on the same methodology. Regardless, the PCA model is second only to the geometric DL network on the “Cross real” experiment in terms of TPR and is very close to the Bull's eye regarding MAE, highlighting the strength of PCA as a shape analysis tool. As for the flattening approaches, the results obtained were ambiguous: while both the Cartesian and Bull's eye model perform similarly in terms of MAE in the experiments including synthetic data (“Sequential Synthetic” and “Sequential Real + Synthetic”), the circumferential approach generalized far more effectively to the real dataset. In fact, the Cartesian grid method was the only model to worsen its accuracy in the realistic LAAs. A possible explanation may be related to the cut-off introduced in the Cartesian grid representation when performing the flattening, which results in the loss of the original mesh topology. This gives rise to a discontinuity when performing the convolution over the flattened mesh, which produces a very prominent cut (see white arrows in Figure 5). As real geometries are far more heterogeneous, the position of the reference 0–360° line marked by the circumflex artery localization might fluctuate more often, which we hypothesize leads to inconsistent learning of the morphological features for the Cartesian method.

On the other hand, the disparity observed between the MAE and the TPR in the real and synthetic cases seems to stem from a distinct distribution of the data. In this respect, the ECAP in the real LAA dataset has a lower μ = 1.664 and a 90th percentile equal to 3.313, whereas the synthetic cases have a μ = 2.266 and a 90th percentile of 4.343. With fewer training data encompassing vertices with ECAP values above the 90th percentile threshold, the model is more prone to fail when confronted with higher ECAP values, leading to far worse TPR (see Table 1). Concerning the gap in the distribution of ECAP values between real and synthetic cases, we observed a prevalence of “Cauliflower” like appendages in the synthetic geometries. These morphologies are characterized by having several lobes, such as cases [2,3,4] in Figure 5. As observed in these three anatomies, the ECAP in these lobes tends to be quite high, probably due to increased blood stasis, which might explain the disparity in the values of ECAP between the two populations. In turn, this is the reason that seems to jeopardize the potential accuracy improvement from including synthetic data.

Moving on to the sequential experiments, all models keep improving as more training data is added, which suggests that further increasing the size of the simulation datasets could be highly beneficial for the overall accuracy. Similarly to the previous experiments, the geometric DL model continues to exhibit superior performance over all the other approaches. This time, it is the PCA-FCN network that struggles the most, as appreciated in the “Sequential Real” and “Sequential Synthetic” experiments, shown in Figure 6, since the amount of initial training data in both of these experiments is well below the 32 principal components that yielded the best results with the PCA model. In fact, the PCA approach was not able to obtain good predictions until the training dataset amounted to about 40 geometries. More interestingly, even though the baseline training dataset already comprised 202 synthetic geometries in the “Sequential Real + Synthetic” experiment, the PCA model did not perform well (i.e., MAE >>1) until a minimum number of real geometries were provided. Finally, the “Sequential Real + Synthetic” experiment suggests that the inclusion of the synthetic data was not of particular help in improving the accuracy of patient-specific LAA. One would expect that, as the amount of real training data increases, the accuracy achieved would eventually exceed that of the model solely trained in the 54 real cases or alternatively, that a similar level of accuracy would be obtained but utilizing a smaller number of real geometries. Neither of these two scenarios held true for any of the models, as accuracy actually worsened overall. Only the geometric DL model managed to achieve accuracy on par with that obtained in the “Sequential Real” experiment, so it appears to have learned more relevant and universal morphological features.

All things considered, the graph-based neural network was superior not only in terms of performance but also regarding the ease of deployment, while the PCA-FCN and flattening models each had their strengths and weaknesses. First, the PCA-FCN model showcased good robustness with regards to real data and it was the faster training model by far. Nonetheless, the need for registration was a major handicap during mesh processing, given that mesh connectivity had to be preserved, which greatly restricted employing tools such as remeshing, vital to avoid mesh quality problems. Not to mention the employed registration itself, which entailed a degree of deformation in the mesh being registered. In regards to flattening, although altogether bypasses the need for registration and template selection, it only succeeded in overcoming the above approach in cases where a very small amount of data was available for training. Besides, UV mapping can not be easily extended to other topologies should we consider including the full LA geometry. All that being said, flattening representations are still very useful for visualization and comparison of large LAA populations. Concerning the geometric DL model, it delivered the best results while working directly over the native form of the data, and using almost an order of magnitude fewer weights than its counterparts. In addition, as no correspondences were required the initial pre-processing was minimal, thus facilitating the editing of the meshes and avoid mesh quality issues. For all these reasons, the graph-based model was chosen for further testing on the second dataset.

### 4.2. Dataset 2—Complete LA Dataset

As aforementioned, an inspection of Figure 7 shows that the distribution of ECAP maps in dataset 2 differs from the synthetic LA dataset (dataset 1). The more complex boundary conditions used in dataset 2 have strengthened the washout in the proximal portion of the LAA. Only in those recesses and cavities in which the inflow fails to reach, the ECAP is higher than in the first dataset. On the other hand, the incorporation of the entire LA geometry during simulation signifies that the ECAP no longer solely depends on the variation of LAA morphology; other anatomical features such as the orientation of the pulmonary veins (García-Isla et al., 2018) will play a role in shaping the variability of the risk index. Despite the added complexity of the second dataset the geometric DL network effectively learned the abstract set of anatomical features related to blood stagnation. Unfortunately, owing to the “black box” nature of neural networks, it is difficult to pinpoint what the model is learning, whether it is a combination of the distance to the ostium along with local curvature on a given bulge or some other arrangement of features that might be challenging for humans to grasp. Although the MAE results effectively tripled in this second dataset relative to dataset 1, it was to be expected given the skewed nature of the data and the extremely high values at given spots.

### 4.3. Limitations and Future Work

Despite the promising results, the presented study has several limitations that must be addressed before it can be of any use in a clinical setting.

First, the choice of the ECAP as a thrombosis risk index may be a subject of contention, since its validity has yet to be proven on the LAA. At first, the ECAP was chosen as it provides a dimensionless scalar field that captures some of the most relevant hemodynamic characteristics related to the formation of thrombi in the LAA, which in turn, allows simplifying the required DL model architecture. Moreover, even though the ECAP index was originally developed in carotid and abdominal aorta fluid models (Di Achille et al., 2014), it has already seen some use in clinical studies exploring device-related thrombus formation in LAA occlusion surgeries (Aguado et al., 2019; Mill et al., 2020). In any case, the underlying mechanisms of thrombus formation in the aforementioned situations always involve some degree of blood stagnation or re-circulation at low velocities that the ECAP should be able to grasp to some extent. Furthermore, there is mounting evidence challenging the utility of standard clinical scores such as the CHAD_2_DS_2_-VASc, which has been long held as the main guide for anticoagulation therapy in AF patients, highlighting the need for more advanced risk indexes accounting for AF-specific factors such as hemodynamic alterations (Siddiqi et al., 2021). In this regard, the geometric DL model could seamlessly extend to 3D data allowing to predict more recently adopted indexes of blood stagnation such as the residence time, which offers an approximate measure of blood stagnation time scale, based on LA flow velocity vector fields (García-Villalba et al., 2021).

On the other hand, the hemodynamic variability arising from the heterogeneous anatomy of the LA was completely neglected when training the network for the sake of simplicity. Nonetheless, since the geometric DL framework does not involve any kind of mesh correspondence it should be fairly trivial to include the complete LA anatomy. Moreover, the network should be capable of learning the ECAP fluctuations caused by factors such as the interaction between the orientation of the pulmonary veins. Yet, an increase in the size of the input graph could render the current local convolution scheme insufficient. In this sense, the network could greatly benefit from widespread approaches in computer vision such as strided convolutions or pooling, aimed at extracting multi-scale features. Nonetheless, although we tested several of the available approaches to construct an encoder-decoder-like architecture such as in Hanocka et al. (2019) and Zhou et al. (2020), for the time being, we have not been able to successfully integrate any of them in the graph-based model. Future work should also be focused on the interpretability of the models, as learning the features that the network is focusing on is a crucial step before being able to deploy the model in a clinical environment.

At the moment, the model is completely agnostic to flow dynamics and distinct boundary conditions that play a key role in the process of thrombogenesis. To address this challenge, we intend on capitalizing on the rapid advances in the field of physics-informed neural networks, with examples such as the study by Pfaff et al. (2021). This may enable the full exploitation by artificial neural networks of the rich Spatio-temporal data available within CFD data, which may pave the way toward the real-time prediction of the full velocity vector field in the LA without the need for hour-long fluid dynamics simulations.

The ground truth from fluid simulations could also be substituted by 4D flow magnetic resonance imaging (MRI), which enables a full non-invasive mapping of the intravascular 3D velocity field over time. Nevertheless, for the time being, most of currently available 4D flow MRI acquisitions employ velocity encodings (V_*enc*_) better suited to higher velocity vessels and continue to suffer from poor signal-to-noise ratio and spatiotemporal resolution (Jiang et al., 2015). As a result, reliable imaging of the LAA flow field is extremely challenging, especially in the proximity of the vessel wall, making it nearly impossible to obtain accurate values of derived hemodynamic indices such as the wall shear stress or the ECAP (Petersson et al., 2012). In this regard, attempts have already been made to tackle said limitations such as the development of Dual-V_*enc*_ acquisition sequences (Callahan et al., 2019) or leveraging CFD simulations to obtain 4D flow super-resolution (Ferdian et al., 2020).

Lastly, to get the full picture of the risk of thrombus formation the inclusion of factors such as endothelial damage/dysfunction could be of particular interest. Scar segmentation in AF patients can be performed automatically by employing neural networks over MRI acquisitions (Li et al., 2020; Yang et al., 2020), allowing detection of left atrium wall fibrosis which is independently associated with LAA thrombogenesis (Guo et al., 2012).

## 5. Conclusion

In the present study, we have successfully leveraged a set of deep learning frameworks to instantaneously predict the ECAP mapping in the LAA solely from its anatomical mesh, effectively skipping the need to run CFD simulations at inference time. All models were effective in a simplified LA model, the graph-based geometrical DL network repeatedly outscoring its competitors. Moreover, this same model exhibited good predictive capability even in more advanced simulations with improved boundary conditions and including the entire LA anatomy. These results could lay the foundation for real-time monitoring of LAA thrombosis risk in the future and open exciting avenues for future preoperative applications and interfaces in which a clinical user could interactively change settings of a left atrial appendage occluder device and instantaneously assess the associated risk of device-related thrombus generation.

## Data Availability Statement

The code to reproduce the experiments of this study is available on github.com/Xtaltec/DL-based-Estimation-of-Thrombotic-Risk. Simulation data, including solver configurations and results, are available under request.

## Ethics Statement

The studies involving human participants were reviewed and approved by the ethics Committee of Rigshospitalet, Copenhagen, Denmark and the ethics committee of Hôpital Haut-Lévêque, Bordeaux, France. The patients/participants provided their written informed consent to participate in this study.

## Author Contributions

XM and OC designed the research and wrote the manuscript. XI, BL, HC, and OD collected and analyzed the data. XM, JM, KJ, CA, HC, BL, XI, OD, RP, and OC did the research and reviewed the manuscript. All authors contributed to the article and approved the submitted version.

## Conflict of Interest

The authors declare that the research was conducted in the absence of any commercial or financial relationships that could be construed as a potential conflict of interest.

## Footnotes

^1^https://www.ansys.com/products/fluids/ansys-fluent

^2^https://es.mathworks.com/products/matlab.html

^3^https://www.paraview.org/

^4^https://keras.io/

^5^https://www.tensorflow.org/

^6^https://github.com/rusty1s/pytorch_geometric

^7^https://pytorch.org/

^8^https://docs.pyvista.org/
