# Supplementary material for: Deep Learning Framework for Real-Time Estimation of in-silico Thrombotic Risk Indices in the Left Atrial Appendage
Source: Front Physiol. 2021 Jun 28;12:694945. doi: 10.3389/fphys.2021.694945 (PMC8274486; doi:10.3389/fphys.2021.694945)
Supplement: Supplementary file 1 [file Data_Sheet_1.PDF]

Supplementary Material

1 SUPPLEMENTARY FIGURES

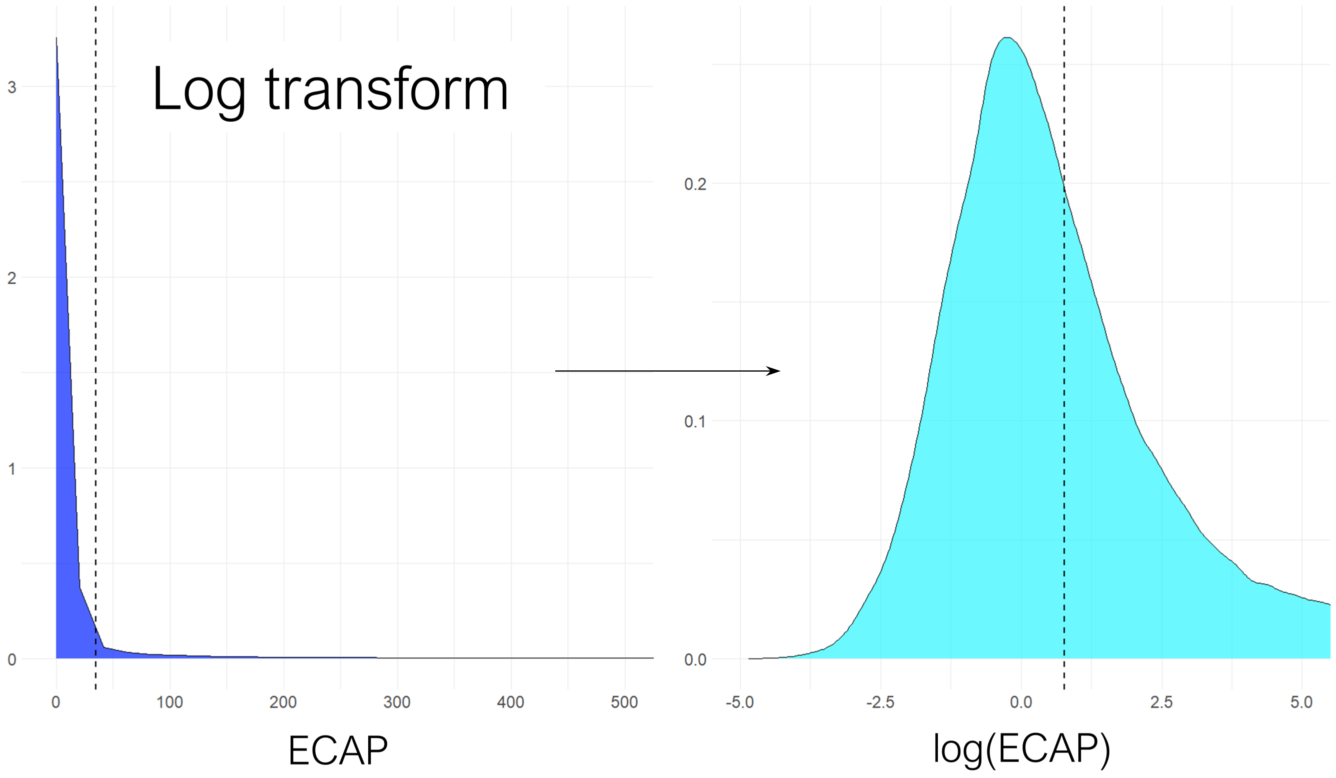

Figure S1. The ground truth ECAP maps from dataset 2 (with patient-specific left atria; LA) were log transformed to obtain a better scaled and more symmetrical distribution before training the neural network. The black dashed line represents the mean of each distribution.

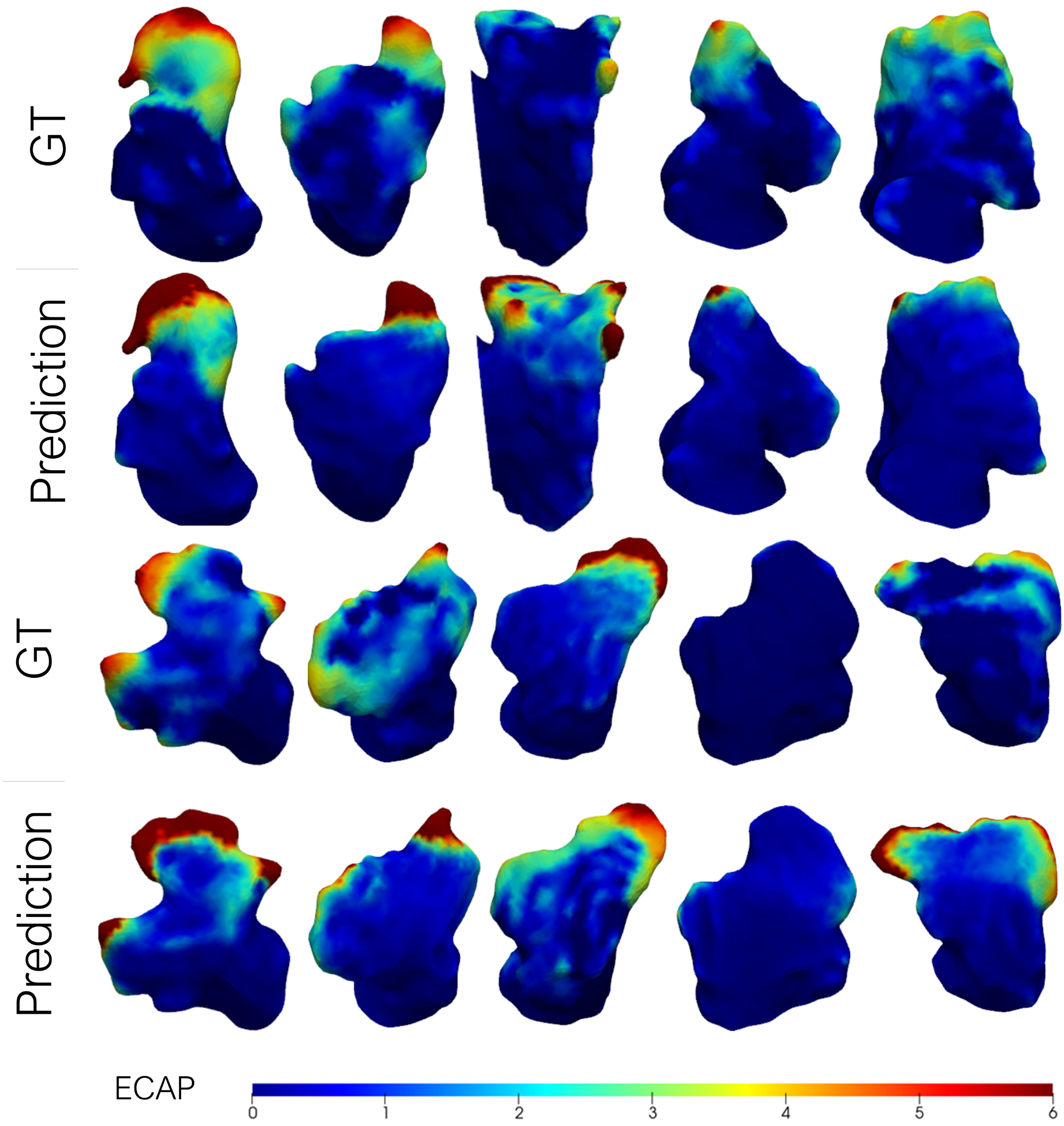

Figure S2. Predicted endothelial cell activation potential (ECAP) maps for the remaining left atrial appendages (LAA) from a fold of the crossvalidation experiment on dataset 2 (i.e. patient-specific LA) alongside the ground-truth (GT) from fluid simulations. Higher ECAP values (in red) indicate a higher risk of thrombus formation.

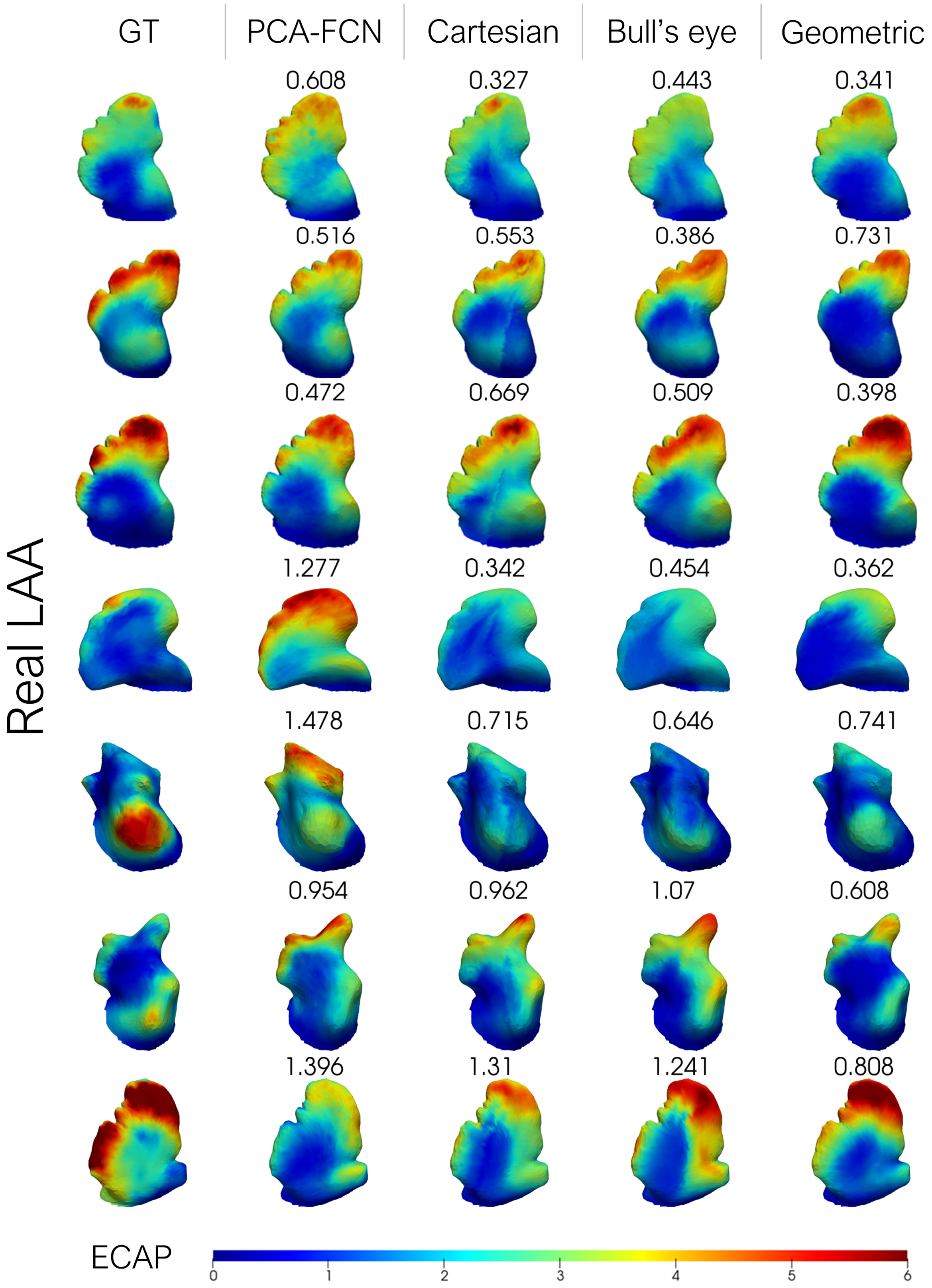

Figure S3. Predictions for the remaining real LAA from one fold of the 'Cross' experiment on dataset 1. From left to right: ground-truth endothelial cell activation potential (ECAP) from fluid simulations; principal component analysis model (PCA-FCN) prediction; Cartesian grid (Cartesian) and bull's eye (Bull's eye) plot prediction; geometric deep learning prediction (Geometric). The mean absolute error is also provided alongside. Higher ECAP values (in red) are linked to a higher risk of thrombus formation.

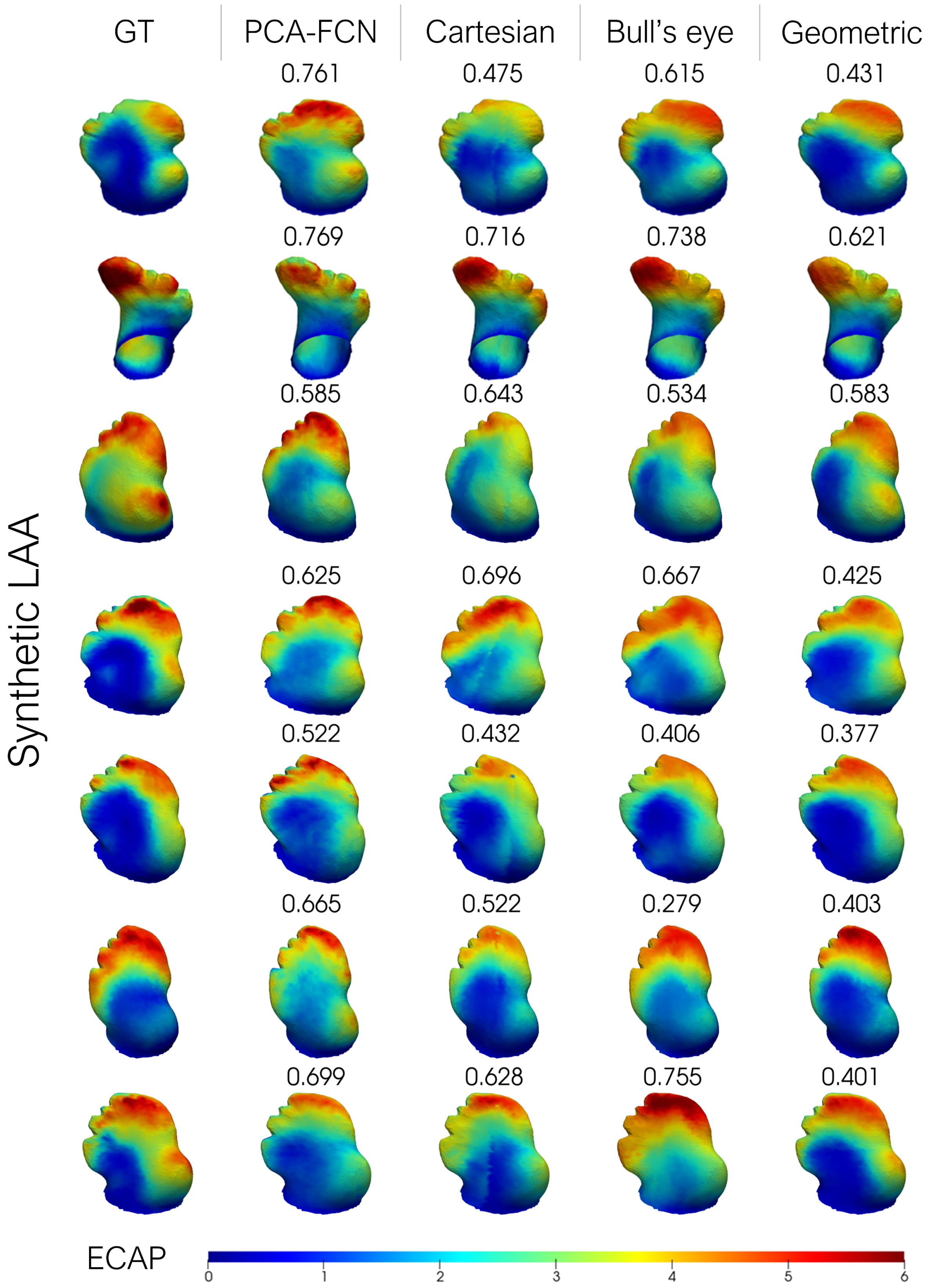

Figure S4. Predictions for the remaining synthetic LAA from one fold of the 'Cross' experiment on dataset 1. From left to right: ground-truth endothelial cell activation potential (ECAP) from fluid simulations; principal component analysis model (PCA-FCN) prediction; Cartesian grid (Cartesian) and bull's eye (Bull's eye) plot prediction; geometric deep learning prediction (Geometric). The mean absolute error is also provided alongside. Higher ECAP values (in red) are linked to a higher risk of thrombus formation.

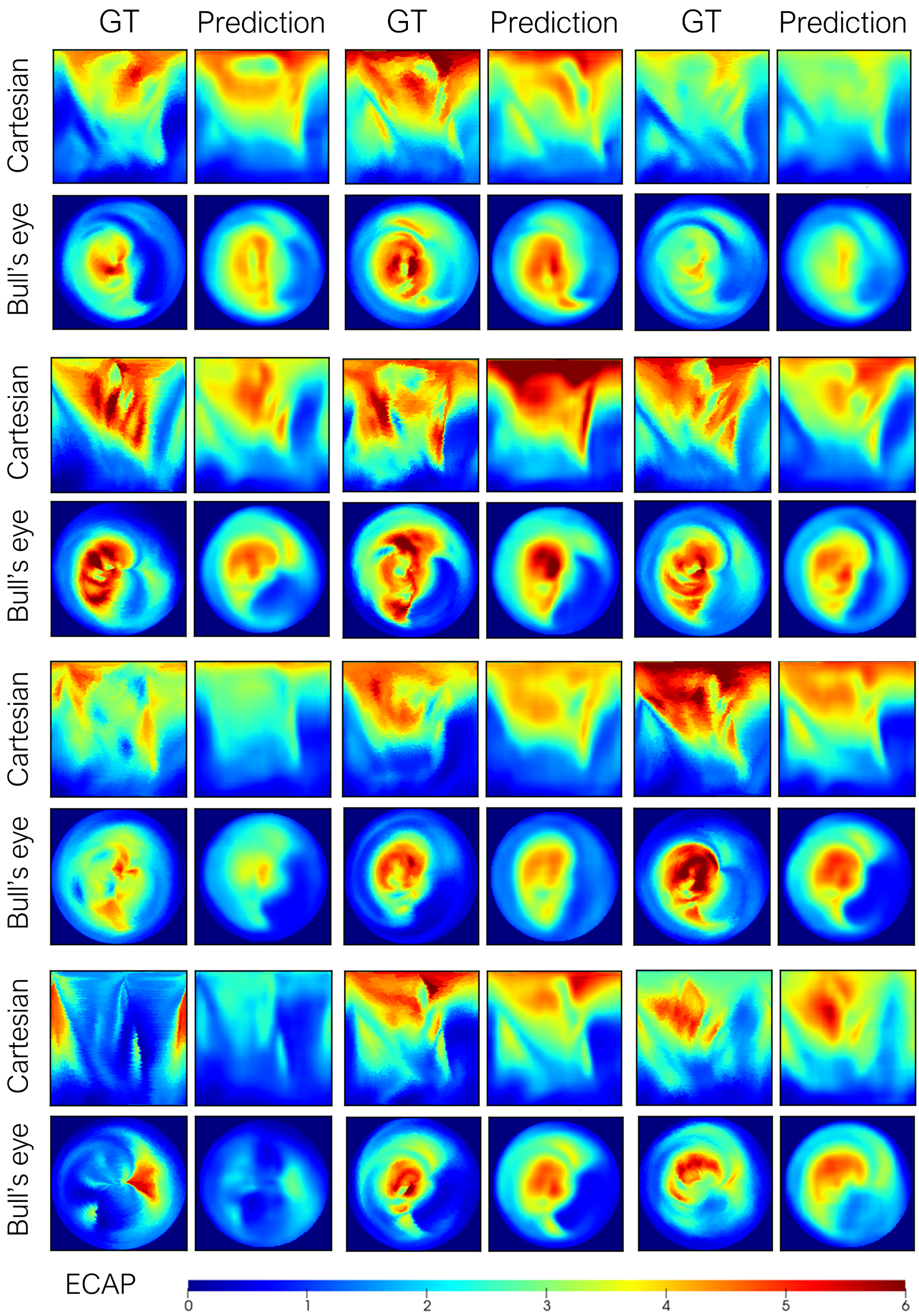

Figure S5. Prediction of the endothelial cell activation potential (ECAP) for the the Cartesian grid and the Bull's eye plot representations associated to the U-Net model. The predictions also pertain to a specific fold of the 'Cross' experiment. ECAP values are colored from low values (blue) to higher than 6 (red).
